# Olfactory ERP-based classification of anosmia and normosmia using machine learning

**DOI:** 10.1016/j.cnp.2026.05.007

**Published:** 2026-06-02

**Authors:** Kwangsu Kim, Thomas Hummel

**Affiliations:** a Smell and Taste Clinic, Department of Otorhinolaryngology, Faculty of Medicine Carl Gustav Carus, Technische Universität Dresden, Fetscherstraße 74, 01307 Dresden, Germany; b Department of Nutritional Science & Food Management, Ewha Womans University, Seoul, Republic of Korea

**Keywords:** Olfactory dysfunction, EEG, Event-related potential, Machine learning, Anosmia

## Abstract

**Objective:**

This study aimed to investigate whether electroencephalogram (EEG) data can be used to classify normosmia and anosmia using machine learning approaches and to examine how classification performance varies across single and combined chemical stimulation conditions.

**Methods:**

EEG signals were recorded from 66 participants (26 normosmic and 40 anosmic) during olfactory stimulation with phenyl ethyl alcohol (PEA), hydrogen sulfide (H₂S), and carbon dioxide (CO₂), presented to the left and right nostrils. EEG data were averaged for each condition, resulting in six chemosensory event-related potential (CSERP) datasets per participant. Three machine learning models—support vector machine (SVM), random forest (RF), and convolutional neural network (CNN)—were used to classify normosmia and anosmia.

**Results:**

Among all single and combined odor conditions, the combination of PEA_Right + H₂S_Left + H₂S_Right achieved the highest performance (ACC = 86.37%, F1 = 0.88, AUC = 0.85). CNN generally outperformed SVM and RF across most conditions. H₂S stimulation consistently provided strong discriminative power, particularly under left-sided presentation.

**Conclusion:**

Machine learning applied to EEG-based CSERPs can effectively distinguish olfactory dysfunction, demonstrating the feasibility of this approach for objective evaluation of olfactory processing.

**Significance:**

These findings suggest that EEG-based machine learning can serve as an objective framework for assessing olfactory function and may contribute to future development of data-driven diagnostic strategies for smell disorders.

## Highlights

• EEG responses to odors were analyzed using machine learning.
• Combined conditions improved classification of normosmia and anosmia.
• Machine learning identified ERP patterns in olfactory processing.

## 1 Introduction

The prevalence of olfactory dysfunction is estimated to be approximately 4–5% in the general population, and it increases with age (Dong et al., 2017; Hummel et al., 2017; Lee et al., 2013; Murphy et al., 2002). Although patients are often unaware of their impairment, previous studies have demonstrated that olfactory function plays a critical role in quality of life (Ahn et al., 2016; Croy et al., 2014; Philpott and Boak, 2014). Furthermore, olfactory dysfunction is a common symptom of neurodegenerative diseases(Doty, 2012; Marin et al., 2018; Son et al., 2021; Yu et al., 2018), making its accurate diagnosis and management essential for maintaining health and well-being.

In clinical practice, olfactory dysfunction is commonly assessed using psychophysical tests such as the Sniffin’ Sticks test(Hummel et al., 1997) or the University of Pennsylvania Smell Identification Test (UPSIT)(Doty et al., 1984). These tests are simple to administer and have been validated in numerous studies(Caminiti et al., 2019; Kim et al., 2020; Rombaux et al., 2007), but they rely on subjective responses and may therefore be influenced by response bias(Hummel et al., 2017). As a valuable addition in clinical practice, electroencephalography (EEG)(Arpaia et al., 2022; Brämerson et al., 2008; Gottschlich and Hummel, 2015; Lötsch and Hummel, 2006; Rombaux et al., 2012) can be used to obtain chemosensory event-related potentials (CSERPs)(Hummel et al., 2017) to assess olfactory function in patients. Compared with psychophysical tests, CSERPs are less affected by subjective bias and can also be useful in medico-legal assessments as well as in patients who may not be able to comply with psychophysical testing, such as young children(Hummel et al., 2017).

Several studies have reported differences in CSERPs between healthy individuals and patients with olfactory dysfunction, particularly anosmia(Arpaia et al., 2022; Brämerson et al., 2008; Huart et al., 2013; Kobal and Hummel, 1998; Lötsch and Hummel, 2006). Previous research has shown that CSERPs are characterized by the N1 (approximately 200–500 ms) and P2 (approximately 600–900 ms) components(Flohr et al., 2015; Wetter and Murphy, 2003). Comparisons of these components between normosmic and anosmic participants revealed that, for olfactory stimuli without trigeminal activation, such as vanillin, CSERPs are typically absent in anosmic patients(Kobal and Hummel, 1998; Wada, 2004). In contrast, trigeminal stimuli can still elicit CSERPs in anosmia(Huart et al., 2013; Kobal and Hummel, 1998; Wada, 2004), although the peak amplitudes could be reduced and the latencies could be prolonged compared with those observed in healthy controls(Huart et al., 2013; Hummel, 2008; Hummel et al., 1996).

Building on these differences in CSERPs, several attempts have been made to evaluate and distinguish normosmic individuals from patients with anosmia(Arpaia et al., 2022; Brämerson et al., 2008; Huart et al., 2013; Kobal and Hummel, 1998; Lötsch and Hummel, 2006; Schriever et al., 2017). However, CSERPs face inherent challenges due to the anatomy of the olfactory system: the primary olfactory cortex is located deep in the brain(Allison, 1954; Gottfried, 2010; Haberly, 1985; Zald and Pardo, 2000), and the olfactory bulb projects broadly in a many-to-many manner to multiple brain regions(Imai, 2014; Imamura et al., 2020). As a result, CSERPs are characterized by a low signal-to-noise ratio and exhibit considerable variability both across individuals and across trials, making them less suitable for strictly phase-locked analyses(Arpaia et al., 2022; Güdücü et al., 2019; Huart et al., 2013). To overcome these limitations, additional approaches such as time–frequency analyses(Huart et al., 2013; Schriever et al., 2017) and entropy-based methods(Güdücü et al., 2019) have been introduced to extract supplementary features directly from the raw EEG data. While these methods can partially address the shortcomings of CSERPs, they require additional computational steps and preprocessing, which may limit their clinical practicality.

With recent advances in machine learning, there has been growing interest in applying these methods to EEG data analysis(Roy et al., 2019; Salehzadeh et al., 2023), including the study of olfaction(Ezzatdoost et al., 2020; Hou et al., 2022; Kasprzak et al., 2024; Lötsch et al., 2019; Peng et al., 2024; Zhang et al., 2021). Most applications so far have focused on healthy participants, for example predicting odor pleasantness(Hou et al., 2022), distinguishing inter-individual differences(Ezzatdoost et al., 2020) in brain responses, or classifying EEG signal patterns elicited by different odors(Peng et al., 2024), and these studies have reported high classification accuracies. However, only a few attempts have been made to use machine learning for classifying EEG data between normosmic and anosmic patients. In particular, studies employing neural network approaches such as convolutional neural networks for this purpose are absent.

Therefore, our purpose in this study was to investigate whether EEG data can be used to classify normosmia and anosmia using machine learning approaches. Rather than relying on single odor stimulation alone, we employed multiple chemical stimuli to examine whether classification performance varies across odors, whether combining odors enhances model performance, and whether trigeminally mediated CSERPs also contribute to classification. To this end, EEG recordings were obtained from patients at the Smell and Taste Clinic, and these data were analyzed with machine learning models to evaluate their ability to differentiate between normosmic and anosmic participants.

## 2 Methods

### 2.1 Participants

We retrospectively analyzed EEG recordings obtained from 66 participants (28 women, 38 men, aged between 21 and 89 years; mean age: 49.70, SD: 17.72). All participants attended the Smell and Taste Clinic at the Department of Otorhinolaryngology of the University of Dresden Medical School because of chemosensory complaints. All tests were performed in accordance with the Declaration of Helsinki on Biomedical Research Involving Human Subjects, and the retrospective study protocol was approved by the Ethics Review Board of the University of Dresden Medical Faculty (process number BO-EK-254062022).

Among the 66 participants, 26 (11 women, 15 men, aged between 21 and 75 years; mean age: 37.88, SD: 15.05) were normosmic, and 40 (17 women, 23 men, aged between 28 and 89 years; mean age: 57.38, SD: 14.98) were anosmic patients (Table 1). The patient groups were determined by the Sniffin’ Sticks test(Hummel et al., 1997). In this test, odors were delivered to participants using felt-tip pens positioned approximately 2 cm in front of both nostrils. The interval between presentations of individual pens within a triplet was approximately 3 s. The olfactory threshold (T) was determined with phenyl ethyl alcohol using a single-staircase procedure with stepwise dilutions across 16 felt-tip pens. Odor discrimination (D) was assessed with a 3-alternative forced choice (3-AFC) task involving 16 pairs of odor stimuli. Odor identification (I) was measured by asking participants to recognize 16 distinct odors, each presented with four verbal descriptors in a forced-choice format. The combined score of threshold (T), discrimination (D), and identification (I) yielded the TDI value, which was used to classify participants as normosmic (TDI ≥ 31) or anosmic (TDI < 16.5)(Hummel et al., 2009). In the anosmia group, the etiology was diverse, including congenital (*n* = 6), post-traumatic (*n* = 7), post-viral (n = 6), idiopathic (n = 6), sinonasal disease (SND, *n* = 14), and Parkinson's disease (n = 1).

**Table 1:** Demographic and clinical information of the normosmia and anosmia groups. This table presents demographic characteristics, Sniffin’ Sticks scores (Threshold, Discrimination, Identification, and total TDI score), and etiological categories in the normosmia and anosmia groups. SND; sinonasal disease.

Categories	Items	Normosmia (*N* = 26)	Anosmia (*N* = 40)	t-value	*p*-value
Demographic Information	Age (years)	37.88	57.38	5.16	<0.001
	Sex (F/M)	11 / 15	17 / 23		
Sniffing Stick	Threshold (T)	10.80	1.13	21.33	<0.001
	Discrimination (D)	12.88	6.03	16.16	<0.001
	Identification (I)	13.54	4.88	15.81	<0.001
	TDI	37.22	12.03	35.01	<0.001
Causes	Congenital	N.A	6		
	Post-traumatic	N.A	7		
	Post-viral	N.A	6		
	Idiopathic	N.A	6		
	SND	N.A	14		
	Parkinson	N.A	1		

### 2.2 Odor preparation and delivery

Two odorants were used for olfactory stimulation: phenyl ethyl alcohol (PEA, 40% *v*/v) and hydrogen sulfide (H₂S, 4 ppm). In addition, carbon dioxide (CO₂, 40%) served as a trigeminal stimulant(Lötsch and Hummel, 2006; Lötsch et al., 2006; Stuck et al., 2006).

Chemosensory nasal stimulation was delivered using a computer-controlled olfactometer (OM2S, Burghart Instruments, Wedel, Germany), which enables administration of gaseous stimuli without concomitant activation of mechanoreceptors or thermoreceptors. Odor pulses (200 ms) were embedded in a continuous airflow (8 L/min) directed into the nasal cavity via a 4-mm inner diameter Teflon™ cannula, positioned approximately 1 cm beyond the nasal valve. The airflow was temperature- and humidity-controlled (36.5 °C, 80% relative humidity), and stimulus onset reached target concentration within <20 ms. For each odorant, 20 stimuli were presented alternately to the left and right nostrils, with an interstimulus interval of 30–40 s to minimize habituation.

### 2.3 Experimental procedure

All EEG measurements were conducted in an air-conditioned room that was darkened to minimize extraneous sensory input. Participants were instructed to keep their eyes open, and breathe through their mouth. They were acoustically shielded (white noise through headphones), their movements were monitored through a video camera system. During EEG recordings subjects had the option to perform a simple tracking task on a computer screen in front of them.

EEG measurements were obtained under six conditions: PEA presented to the left and right nostrils, H₂S presented to the left and right nostrils, and CO₂ presented to the left and right nostrils. Each condition comprised 20 trials. In total, approximately 120 trials were conducted for each participant.

### 2.4 EEG recording and preprocessing

EEG activity was recorded from five standard sites of the international 10–20 system (Cz, C3, C4, Fz, and Pz), with linked earlobes (A1 and A2) as reference. Eye-blink activity was monitored at Fp2. Continuous EEG was sampled at 250 Hz with a band-pass filter of 1–30 Hz. Stimulus-locked epochs were extracted from −200 to 1000 ms relative to stimulus onset, with the −200 to 0 ms interval serving as baseline. Baseline correction was applied to all epochs. Epochs containing eye blinks (>50 μV at Fp2) or other artifacts (e.g., high-frequency muscle activity) were excluded after visual inspection by a trained observer. Across all participants, the minimum number of artifact-free segments available for averaging was six.

### 2.5 Event-related potentials

After EEG preprocessing, data were averaged for each condition. Consequently, each participant had six CSERP datasets corresponding to the six conditions (“PEA_Left,” “PEA_Right,” “H₂S_Left,” “H₂S_Right,” “CO₂_Left,” and “CO₂_Right”). The CSERPs were visually inspected by an experienced researcher to confirm the presence of typical waveform patterns across conditions(Wetter and Murphy, 2003).

### 2.6 Classification based on machine learning

To investigate whether EEG data could be used to classify normosmic and anosmic participants, we applied three machine learning approaches: support vector machine (SVM), random forest (RF), and convolutional neural network (CNN).

The data were collected in a clinical setting, where patients underwent EEG measurements during their hospital visits. Given this context, we aimed to minimize additional preprocessing and feature engineering. Accordingly, only band-pass filtering and simple averaging were applied prior to analysis. For model training, the full Cz EEG signal from 0 to 1000 ms after stimulus onset was used as input.

To increase the amount of available information for classification and to account for potential asymmetries in olfactory sensitivity between nostrils, we constructed various combinations of odor conditions and examined whether such combinations improved classification performance. The combinations included single conditions (PEA_Left, PEA_Right, H₂S_Left, H₂S_Right, CO₂_Left, CO₂_Right), bilateral conditions (PEA_Left + PEA_Right, H₂S_Left + H₂S_Right, CO₂_Left + CO₂_Right), and multi-odor conditions such as PEA_Left + H₂S_Left, PEA_Right + H₂S_Right, PEA_Left + H₂S_Left + CO₂_Left, PEA_Right + H₂S_Right + CO₂_Right, PEA_Right + H₂S_Left + H₂S_Right, PEA_Left + PEA_Right + H₂S_Left + H₂S_Right, and the full combination of all six odor conditions. In particular, the combination PEA_Right + H₂S_Left + H₂S_Right was selected for additional testing because it represented one of the odor conditions with higher classification accuracy.

All three models (SVM, RF, CNN) were optimized with a grid-search procedure combined with five-fold cross-validation. For each parameter combination and fold, performance was quantified using accuracy (ACC), the area under the receiver operating characteristic curve (AUC), and the F1-score. The final model for each algorithm was chosen based on cross-validated performance across these metrics. The same evaluation protocol was applied to every odor condition and to the predefined odor combinations. Performance comparisons between models and odor conditions were conducted descriptively based on cross-validated metrics averaged across folds.

For SVM, SVM models were trained using different kernel functions (linear, radial basis function, and polynomial). EEG features were z-score normalized prior to training, and hyperparameters were optimized with grid search under the five-fold cross-validation framework. Model selection was based on cross-validated performance metrics (ACC, AUC, F1-score).

For RF, RF models were constructed as ensembles of decision trees. The number of estimators, leaf size, and the number of predictors sampled at each split were varied during model optimization. Common feature selection strategies, such as using the square root or half of the available predictors, were tested, and surrogate splitting was also considered. Hyperparameters were tuned by grid search with five-fold cross-validation, and the best-performing configuration was selected according to cross-validated performance.

For CNN, the one-dimensional Cz EEG signal (0–1000 ms) was reshaped into a time × channel tensor and analyzed with one-dimensional convolutions implemented as 2D kernels of size k × 1. The network consisted of two convolutional blocks, each including a convolution layer, rectified linear unit activation, max pooling along the time axis, and dropout. These blocks were followed by a fully connected layer, an additional nonlinearity, and a final two-unit softmax output layer for binary classification. Training was performed with the Adam optimizer, a moderate mini-batch size, and a limited number of epochs. Hyperparameters such as the number of filters, kernel size, pooling size, number of dense units, dropout rate, and learning rate were optimized through grid search within the same five-fold cross-validation framework.

### 2.7 Statistics

The significance in statistics was marked as * for *p* < 0.05, ** for *p* < 0.01, and *** for *p* < 0.001. Between-group comparisons of continuous variables (e.g., age, TDI, threshold, discrimination, and identification scores) were performed using two-tailed independent-samples *t*-tests, and results are reported with t values, degrees of freedom (df), *p* values and Cohen's d as a measure of effect size. Sex distribution between normosmic and anosmic groups was compared using a chi-square test, with χ^2^ values and p values reported. All statistical tests were two-tailed.

### 2.8 Software

Electrophysiological data were analyzed using MATLAB 2024b, in conjunction with toolboxes that included EEGLAB.(Delorme and Makeig, 2004) MATLAB was also used for statistical analysis.

## 3 Results

### 3.1 Demographic and psychophysiological differences between normosmia and anosmia

Demographic and psychophysical characteristics of the participants are summarized in Table 1. The anosmic group was significantly older (57.38 ± 14.98 years) than the normosmic group (37.88 ± 15.05 years; t₆₄ = 5.16, p < 0.001, d = 1.15). As expected, all Sniffin’ Sticks subscores were markedly lower in the anosmic group compared with the normosmic group: threshold (t₆₄ = 21.33, p < 0.001, d = 4.77), discrimination (t₆₄ = 16.16, p < 0.001, d = 3.61), identification (t₆₄ = 15.81, p < 0.001, d = 3.54), and total TDI score (t₆₄ = 35.01, p < 0.001, d = 7.83). Sex distribution did not differ significantly between groups (χ^2^ = 0.21, *p* > 0.05). These results confirm the expected group differences, consistent with the clinical characteristics of anosmia.

### 3.2 Classification between normosmia and anosmia by EEG

**Fig. 1:**
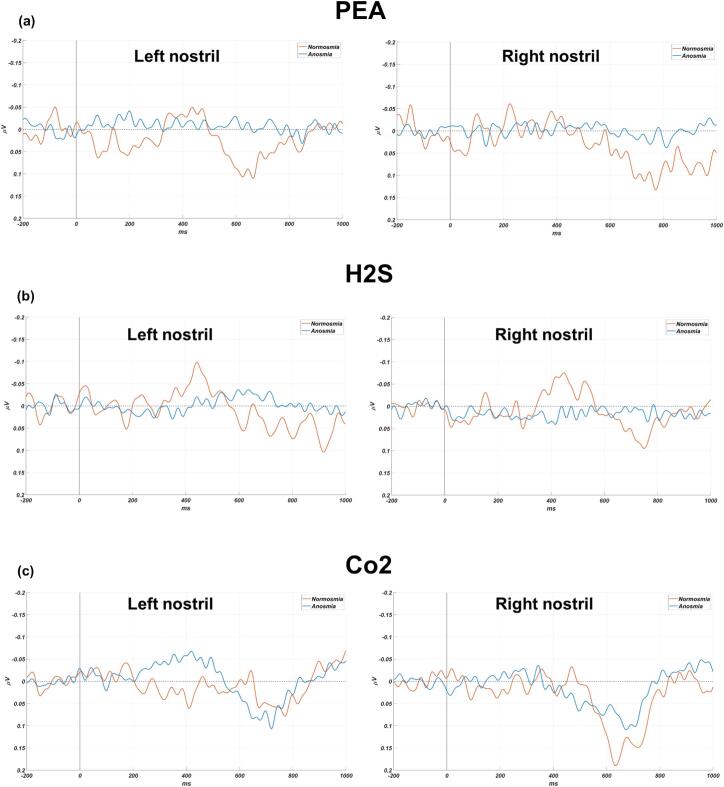
Representative CSERPs for a selected normosmic and a selected anosmic participant under the PEA (left, right), H₂S (left, right), and CO₂ (left, right) conditions. The same participant was selected for all conditions within each group. The orange line represents the normosmic participant, and the blue line represents the anosmic participant. CSERP waveforms are shown from the Cz channel, with the y-axis in μV (reversed, negative plotted upwards) and the x-axis from −200 to 1000 ms. (a) PEA (left, right). (b) H₂S (left, right). (c) CO₂ (left, right). (For interpretation of the references to colour in this figure legend, the reader is referred to the web version of this article.)

Differences between the normosmic and anosmic groups were also observed in CSERP responses across odor conditions. Representative CSERPs from a selected participant of each group (Fig. 1) showed distinct waveform patterns over time. To quantify these differences, we conducted classification analyses using three machine learning models (SVM, RF, and CNN) to differentiate normosmia and anosmia (Table 2).

**Table 2:** Classification performance between normosmia and anosmia using SVM, Random Forest, and CNN models. This table presents classification accuracy (ACC), F1-score, and area under the curve (AUC) across odor conditions (PEA, H₂S, and CO₂) and their combinations. The highest accuracy was obtained for the combination of PEA_Right, H₂S_Left, and H₂S_Right, with CNN showing the best overall performance (ACC = 86.37%). ACC, accuracy; AUC, area under the curve; CNN, convolutional neural network; SVM, support vector machine.

Classification between normosmia vs. anosmia									
Odor condition	Model								
	SVM			Random forest			CNN		
	ACC	F1-score	AUC	ACC	F1-score	AUC	ACC	F1-score	AUC
PEA_Left	65.05%	0.73	0.64	62.20%	0.74	0.52	72.64%	0.79	0.68
PEA_Right	66.48%	0.73	0.72	66.81%	0.75	0.68	77.25%	0.78	0.77
H₂S_Left	80.33%	0.85	0.77	84.73%	0.87	0.81	83.30%	0.87	0.87
H₂S_Right	75.49%	0.78	0.80	72.97%	0.80	0.78	81.87%	0.84	0.77
CO₂_Left	66.48%	0.78	0.56	60.55%	0.70	0.53	74.29%	0.77	0.73
CO₂_Right	74.51%	0.80	0.75	64.73%	0.74	0.71	72.53%	0.78	0.73
PEA_Left + PEA_Right	65.16%	0.74	0.71	69.67%	0.78	0.74	78.90%	0.84	0.75
H₂S_Left + H₂S_Right	80.22%	0.84	0.88	83.30%	0.87	0.83	81.76%	0.86	0.80
CO₂_Left + CO₂_Right	63.63%	0.73	0.60	68.13%	0.75	0.61	72.86%	0.79	0.68
PEA_Left + H₂S_Left	77.47%	0.83	0.78	81.87%	0.84	0.81	77.47%	0.81	0.70
PEA_Right + H₂S_Right	79.01%	0.82	0.88	71.21%	0.77	0.74	83.30%	0.87	0.81
PEA_Left + H₂S_Left + CO₂_Left	79.12%	0.79	0.74	77.03%	0.81	0.86	77.03%	0.80	0.72
PEA_Right + H₂S_Right + CO₂_Right	77.25%	0.81	0.86	66.48%	0.76	0.80	82.09%	0.83	0.78
PEA_Right + H₂S_Left + H₂S_Right	78.90%	0.83	0.81	84.73%	0.88	0.85	86.37%	0.88	0.85
PEA_Left + PEA_Right + H₂S_Left + H₂S_Right	80.11%	0.83	0.83	80.44%	0.85	0.77	86.26%	0.89	0.84
PEA_Left + PEA_Right + H₂S_Left + H₂S_Right + CO₂_Left + CO₂_Right	85.05%	0.88	0.84	76.04%	0.82	0.76	79.01%	0.84	0.70

Among all conditions and models, the combination PEA_Right + H₂S_Left + H₂S_Right showed the highest observed classification performance (ACC = 86.37%, F1 = 0.88, AUC = 0.85). This combination was selected based on the single-odor conditions that showed the top three accuracies.

Across the three models, CNN tended to provide better performance in 10 out of 16 conditions, whereas RF and SVM each yielded the best performance in 3 conditions.

When lateralized conditions were systematically compared (PEA, H₂S, CO₂, and their left- vs right-sided combinations), PEA consistently showed higher classification accuracy under right-sided stimulation, whereas H₂S performed better under left-sided stimulation. For CO₂ and mixed odor combinations, the superiority of one side over the other varied depending on the model, indicating no consistent lateral dominance.

For single odor conditions, the best performance was obtained with H₂S_Left (RF, ACC = 84.73%, F1 = 0.87, AUC = 0.81), highlighting the strong discriminative power of H₂S stimulation. H₂S_Right also yielded high accuracy (CNN, ACC = 81.87%, F1 = 0.84, AUC = 0.77), further supporting the robustness of H₂S in separating normosmia from anosmia. In comparison, PEA produced lower accuracies, with the right-sided condition outperforming the left (PEA_Right, CNN, ACC = 77.25%, F1 = 0.78, AUC = 0.77; PEA_Left, CNN, ACC = 72.64%, F1 = 0.79, AUC = 0.68). CO₂ conditions showed more variable outcomes: CO₂_Left performed best with CNN (ACC = 74.29%, F1 = 0.77, AUC = 0.73), while CO₂_Right yielded its highest accuracy with SVM (ACC = 74.51%, F1 = 0.80, AUC = 0.75).

For bilateral odor conditions, H₂S_Left + H₂S_Right achieved the strongest results (RF, ACC = 83.30%, F1 = 0.87, AUC = 0.83), confirming the robustness of H₂S-based classification. PEA_Left + PEA_Right also showed improved performance compared to single PEA conditions (CNN, ACC = 78.90%, F1 = 0.84, AUC = 0.75). By contrast, combining CO₂ from both sides did not enhance accuracy, with CNN yielding only moderate results (ACC = 72.86%, F1 = 0.79, AUC = 0.68).

For multi-odor combinations, strong performances were again observed. PEA_Left + PEA_Right + H₂S_Left + H₂S_Right achieved one of the top accuracies (CNN, ACC = 86.26%, F1 = 0.89, AUC = 0.84), second only to PEA_Right + H₂S_Left + H₂S_Right. Among smaller combinations, PEA_Right + H₂S_Right reached high performance (CNN, ACC = 83.30%, F1 = 0.87, AUC = 0.81), while PEA_Left + H₂S_Left showed more stable but slightly lower results (RF, ACC = 81.87%, F1 = 0.84, AUC = 0.81). Adding CO₂ yielded mixed outcomes: PEA_Right + H₂S_Right + CO₂_Right improved accuracy (CNN, ACC = 82.09%, F1 = 0.83, AUC = 0.78), whereas PEA_Left + H₂S_Left + CO₂_Left showed lower performance (SVM, ACC = 79.12%, F1 = 0.79, AUC = 0.74). Finally, when all six odors were combined, SVM achieved strong results (ACC = 85.05%, F1 = 0.88, AUC = 0.84), although this was not superior to the best four-odor subset.

Our results indicate that the highest accuracies were obtained with H₂S-based conditions, and that combining H₂S with other odors further improved classification, yielding the best overall performance. This suggests that odor combinations provide additional discriminative information for differentiating normosmia and anosmia, with CNN generally outperforming RF and SVM, particularly for complex odor sets.

## 4 Discussion

We found that EEG data obtained under combinations of odor conditions enhanced classification performance between normosmia and anosmia. In particular, conditions involving H₂S, either alone or combined with other odors, tended to yield the highest accuracies. These findings suggest that odor combinations provide additional discriminative information beyond single odors, thereby improving the ability of machine learning models to differentiate olfactory function groups. Thus, the enhanced classification observed for odor combinations likely builds on underlying neural response patterns that are sensitive to both odor quality and group differences in olfactory processing.

Among the chemical stimulation conditions, the most pronounced classification performance was observed for H₂S. These findings suggest that classification performance may depend on the chemical stimulus employed.

In our study, combining multiple chemical stimuli improved classification performance between normosmia and anosmia compared with using single stimuli alone. The last three conditions in Table 2, each involving more than two stimuli, tended to yield the highest accuracies, with values of 86.37%, 86.26%, and 85.05%, respectively. These findings indicate that employing multiple chemical stimuli in EEG paradigms can enhance the ability of machine learning models to detect CSERP-related differences in olfactory processing. Moreover, the peak accuracy of 86.37% observed in our study exceeds that reported in previous research(Güdücü et al., 2019; Huart et al., 2013; Schriever et al., 2017) on normosmia–anosmia classification, suggesting that our approach contributes to automating the identification of ERP patterns related to olfactory processing.

Several points should be considered when interpreting the results of the present study. First, the number of artifact-free trials was relatively low (≥6 in some participants). Although meaningful classification results were obtained, the limited number of trials may have affected the signal-to-noise ratio and, consequently, the reliability of the findings. Therefore, the results should be interpreted with caution. Second, the present study primarily focused on data from the Cz electrode. While Cz has been widely used in olfactory ERP research and clinical applications due to its stability and reproducibility (Arpaia et al., 2022; Brämerson et al., 2008), it is not anatomically closest to olfactory-related brain regions. It is possible that electrode locations closer to the olfactory bulb (e.g., subfrontal regions) or to primary olfactory cortex areas (e.g., temporal regions) may provide improved classification performance. Furthermore, recent approaches such as electrobulbogram (EBG) recordings (Iravani et al., 2020), which more directly capture olfactory bulb activity, may offer additional advantages for classification tasks. However, these approaches were beyond the scope of the present study and should be considered in future research.

In conclusion, unlike previous studies that primarily focused on single-stimulus paradigms or relied on conventional CSERP analyses, our study demonstrated that combining multiple chemical stimuli with machine learning approaches provides very good classification of normosmia and anosmia. While additional validation with larger and more diverse datasets will be necessary, the present results demonstrate the feasibility of using machine learning to objectively identify CSERP patterns associated with olfactory processing.

## Author contribution

KK and TH conceived and designed the study. KK performed the data analysis, interpreted the data, and prepared the figures and original draft of the manuscript. TH critically revised the manuscript and supervised the study.

## Declaration of competing interest

The authors declare no competing interests.

## Data Availability

The datasets generated during and/or analyzed during the current study are available from the corresponding author on reasonable request.
